# Self-Aware Artificial Coiled Yarn Muscles with Enhanced Electrical Conductivity and Durability via a Two-Step Process

**DOI:** 10.3390/polym15030552

**Published:** 2023-01-20

**Authors:** Yongqi Gong, Wanyi Chen, Jianyang Li, Shun Zhao, Luquan Ren, Kunyang Wang, Bingqian Li

**Affiliations:** 1 Key Laboratory of Bionic Engineering, Ministry of Education, Jilin University, Changchun 130022, China; 2 State Grid Jilin Electric Power Co., Ltd., Changchun 130022, China

**Keywords:** artificial muscle, self-aware, coiled yarn, soft actuation, integration

## Abstract

Muscles are capable of modulating the body and adapting to environmental changes with a highly integrated sensing and actuation. Inspired by biological muscles, coiled/twisted fibers are adopted that can convert volume expansion into axial contraction and offer the advantages of flexibility and light weight. However, the sensing-actuation integrated fish line/yarn-based artificial muscles are still barely reported due to the poor actuation-sensing interface with off-the-shelf fibers. We report herein artificial coiled yarn muscles with self-sensing and actuation functions using the commercially available yarns. Via a two-step process, the artificial coiled yarn muscles are proved to obtain enhanced electrical conductivity and durability, which facilitates the long-term application in human-robot interfaces. The resistivity is successfully reduced from 172.39 Ω·cm (first step) to 1.27 Ω·cm (second step). The multimode sense of stretch strain, pressure, and actuation-sensing are analyzed and proved to have good linearity, stability and durability. The muscles could achieve a sensitivity (gauge factor, GF) of the contraction strain perception up to 1.5. We further demonstrate this self-aware artificial coiled yarn muscles could empower non-active objects with actuation and real-time monitoring capabilities without causing damage to the objects. Overall, this work provides a facile and versatile tool in improving the actuation-sensing performances of the artificial coiled yarn muscles and has the potential in building smart and interactive soft actuation systems.

## 1. Introduction

Twisting is a genius invention of nature that imparts enhanced mechanical properties to non-functional fibers by increasing their compactness, which could be found in widespread molecules (DNA, protein) [1,2], plants (vine) [3,4] and animals (snail) [5,6]. Inspired by the twisting structures, numerous artificial muscles with twisted or coiled structures have been proposed with fish line/yarn fibers [7,8,9]. The commonly employed fish line/yarn fibers are thermal-responsive materials that could generate reversible transformation by altering their long highly oriented polymer molecules. For instance, the thermal contraction of nylon 6,6 fibers can reach values similar to those of NiTi shape memory wire [10], but this contraction does not meet the requirements of the majority of practical applications. The introducing of twisting into those fibers greatly enhances the contraction effect, and the actuation properties of the resulted muscles could be regulated via the degree of twisting and the tightness of the resulting coil [11]. In comparison to other artificial muscles e.g., dielectric elastomers, conductive polymers, hydrogels and shape memory polymers, artificial coiled yarn muscles offer unparalleled advantages regarding low voltage, reversibility, high specific energy density and economy [12,13,14]. In particular, with the optimization of materials, structures and stimuli, recent artificially curled muscles have shown higher performance in terms of actuation stroke, speed, response time, specific energy density and specific work output [15,16].

With the developing trend of miniaturization, light weight and intelligence of soft robots and artificial muscles, actuation-sensing integration is increasingly emerging as a focus of the artificial muscles [17,18,19]. Currently, the actuation-sensing integration of artificial muscles is mainly divided into two categories. The first category is integrating flexible sensors into artificial muscles, where the actuation and sensing rely on independent modules and usually generate unsatisfied material interface [20,21]. One typical investigation was reported by Zhao’s group, which employed a stretchable optical waveguide as a strain-sensing component of a prosthetic hand to perceive the shape and softness of an object [21]. The second approach is employing the intrinsic sensing properties from the active materials, which can significantly reduce complexity of the integrated systems and provide better sensing-actuation interfaces [22,23,24]. Shi’s group proposed an integrated sensor-actuator by combining nanocarbon black/polylactic acid composites with bioinspired gradient micro-gap structures with 4D printing method [23]. Under the stimulation of heat, the integrated sensor-actuator can actively touch objects and self-sense the touching state through the resistance changes.

For fiber-based artificial muscles, the primary approach is to adopt core-shell structure [25,26], coating [27,28] and electrostatic spinning method [29,30]. For instance, Liu’s group presented twisted elastomer fiber artificial muscles by using twisted natural rubber fiber coated with a buckled carbon nanotube sheet, in which the twisted natural rubber fiber can be electrothermally actuated by entropic elasticity and the buckled carbon nanotube sheet can transmit electric current by thermo-piezoresistive effect [31]. Di’s group reported nanofiber-interfaced triple-layered coaxial structures by wrapping MXene/CNT thin sensing sheath around CNT/elastomer actuation core, which builds a dielectric capacitor and enables sensitive touchless perception [26]. However, there are barely reports on fish line/yarn-based sensor-actuator so far. The main reason is that the raw material for fishing line/yarn is often off the shelf filament, and traditional post-treatment methods often suffer from low conductivity and durability problems.

Here, we propose a two-step method for fabricating self-aware artificial coiled yarn muscles. By leveraging the difference in morphology between the planar structure of carbon black and the fibrous form of carbon nanotubes, we achieve superior penetration into the surface and interior to improve the electrical conductivity and durability of the yarn. The artificial coiled yarn muscles can perceive multi-somatosensory tactile signals including stretching strains and pressure and can monitor the electrothermal actuation process in real time. We provide a facile and versatile method in fabricating self-aware artificial coiled yarn muscles, and intrinsic actuation-sensing properties from the active materials can reduce the complexity of intelligent structures and systems, thereby facilitating the development of the field of flexible wearable devices, soft robotics, etc.

## 2. Materials and Methods

### 2.1. Fabrication of the Self-Aware Artificial Coiled Yarn Muscles

**Figure 1:**
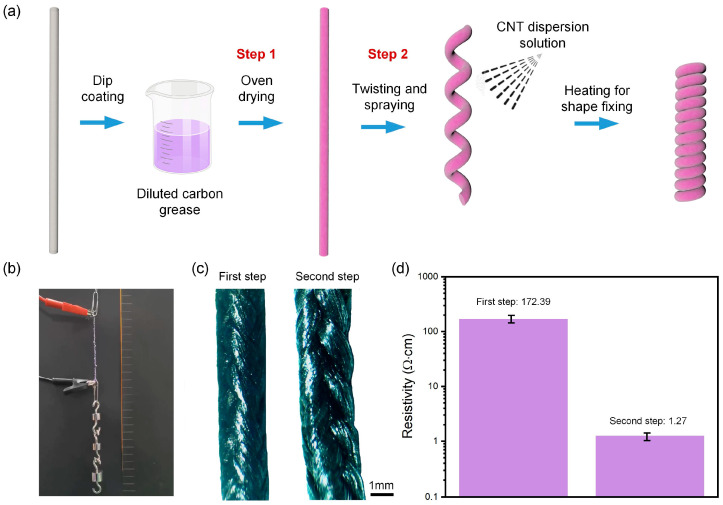
Fabrication and characterization of the artificial coiled yarn muscles via the two-step method. (**a**) The fabrication process of the self-aware artificial coiled yarn muscles; (**b**) The picture of the artificial coiled yarn muscles after the two-step process; (**c**) The microscopic optical images of muscle after the first step (left) and second steps (right); (**d**) The resistivity of the muscle after the first step and second steps.

The fabrication process of the self-aware artificial coiled yarn muscles is shown in Figure 1a. The commercially available nylon yarn fiber is first dip coated in diluted carbon grease with acetone, and oven drying in 50 °C for 12 h to make the carbon black uniformly adhere to the fiber surface or penetrate into the interior. Then, apply a torque to the dip-coated yarn fiber to form coiled muscles. It is worth noting here that the twisting process caused some of the carbon black to fall off due to the friction of the fibers, resulting in uneven conductivity of the resulting artificial muscle. Therefore, we adopted the second step, ink-jetting carbon nanotubes (CNTs) dispersion solution on the yarn fibers during the twisting process. Then, the obtained self-aware artificial muscles are heated to 80 °C to fix the coiled shape. This method not only fills the carbon black particles that fall off during the twisting process, but also provides a secondary filling of the original conductive particles relying on the morphology differences to enhance the overall conductivity and stability. During the curling process, the weight of the balance weights and the speed of curling have a closely related effect on the curling uniformity of the artificial muscle. In our experiments, we select the optimized processing parameters to achieve the best curl effect.

### 2.2. Characterization

In Figure 1b, the picture of the artificial coiled yarn muscles after the two-step process is taken by Nikon Camera (Nikon D5300, Tokyo, Japan). The microscopic optical images after the first and second steps of processing were characterized by using an optical microscope (Nikon LV 100NPOL, Tokyo, Japan) (Figure 1c). The images show that the diameter of the fibers after the second processing step is larger than that after the first processing step, proving that the second treatment brings a thicker coated conductive layer. High precision digital multimeter (ADCMT 7351E, Saitama, Japan) and vernier calipers are used for resistance measurement. Their resistivity results are shown in the Figure 1d, which demonstrates that after the second treatment step, there are more uniform adhesion of conductive particles and higher conductivity on the fiber surface. In addition, we found that after the secondary treatment, the phenomenon of particle shedding was significantly improved. The weight of the artificial muscle after actuation for ten cycles was basically no change.

### 2.3. Development of the Real-Time Signal Detection System in the Muscle Actuation Process

**Figure 2:**
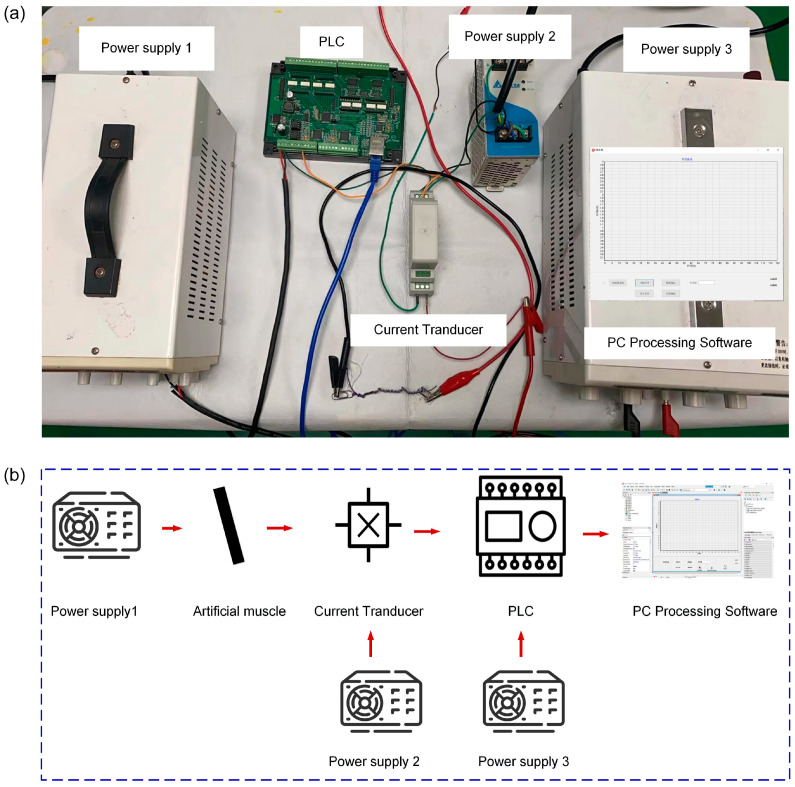
Real-time signal detection system. (**a**) Experimental setups of the signal detection system; (**b**) Schematic diagram of the signal detection system.

The controlling system including three power supplies, a current transducer and a PLC control board is developed to detect the signals during the actuation process (Figure 2). The current transducer is utilized to detect the current changes in the actuation process of artificial muscle, and the self-developed software is utilized for transiting the current changes to resistance changes under specific voltages. One of these three power supplies is employed to actuate the artificial muscle through Joule heating, and the rest two power supplies are used to power the current transducer and a PLC control board, respectively. In this method, the real-time resistance changes in the actuation process could be monitored through intuitive curve display on the computer.

### 2.4. Statistical Analysis

Each test of lifting experiments was repeated 10 valid times under same conditions for further analysis and comparison. The results are showed as mean ± standard deviation. Statistical significance was tested using ANOVA (single factor) by SPSS 25.0 software (IBM, Armonk, NY, USA). Probability values of *p* < 0.05 were considered statistically significant, and all data are presented at a *p* < 0.05 significance level unless otherwise stated.

## 3. Results and Discussion

### 3.1. Multimode Sense of Stretching Strain and Pressure

**Figure 3:**
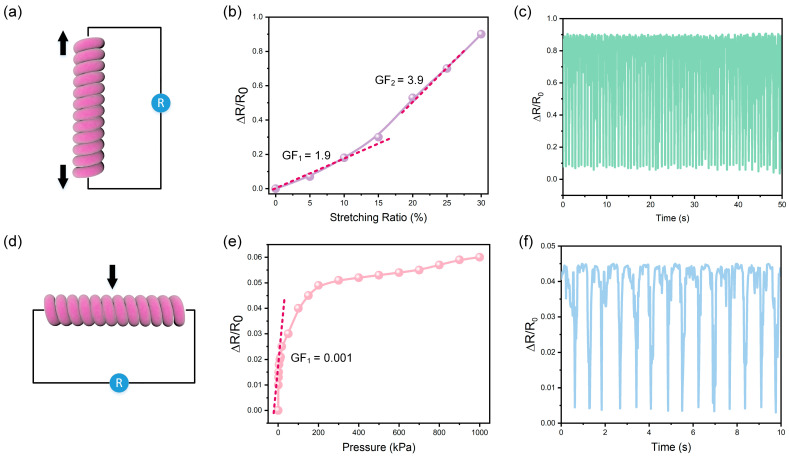
Stretching strain and Pressure perceptive properties. (**a**) Schematic illustration of the working mechanism for the stretching perception; (**b**) The sensitivity of the stretching perception; (**c**) Resistance variation of stretching cycle tests; (**d**) Schematic illustration of the working mechanism for the pressure perception; (**e**) The sensitivity of the pressure perception; (**f**) Resistance variation of pressing cycle tests.

The piezo-resistance properties of the artificial coiled yarn muscles by stretching and pressing were investigated. The fiber was stretched with different strain ratio via a self-made tensioning device, and the corresponding resistance changes was recorded via digital multimeter software (Figure 3a). The sensitivity (gauge factor, GF) of the artificial muscle is calculated as follows.

GF = (∆R/R_0_)/ε, (1)

where ∆R is the changed resistance in the stretching process, R_0_ is initial resistance, and ε is the strain ratio of the artificial coiled yarn muscles. The resistance increases linearly with the increase of pressure in both the low strain region (0 to 10%) and the high-pressure region (10% to 30%) but with different slopes (Figure 3b). These results can be attributed to the increasing distance between the conductive particles in the stretching process. However, when stretched to a certain threshold, the connection between particles gradually loses, thus leading to a decrease in sensitivity or even failure. In addition, excessive stretching ratios can lead to unwinding of the coiled structure. Thus, the maximum strain ratio was settled at 30%. To test the sensing stability and durability of this artificial muscle, 50 cycles of stretching-release tests were performed under a stretching ratio of 30%, indicating good performance of the artificial muscle (Figure 3c).

In addition, the fiber was compressed with different pressures via a self-made pressing device. The pressure perception of the artificial coiled yarn muscle was shown in Figure 3d. The resistance change has a linear relationship in the pressure range from 0 to 20 kPa and shows a plateau relationship with further increasing pressure. By replacing the strain in the sensitivity Equation (1) with the pressure, the sensitivity under pressures could be obtained (Figure 3e). Overall, the sensitivity of artificial muscles is much lower than the stretch sensitivity, which may be due to the fact that the particles are already close enough to each other in the cross-sectional direction. When a certain critical value is reached, the interface between the particles achieves complete contact, resulting in a sensitivity of almost zero at this point. Although its sensitivity is reduced compared to the stretching direction, it still has good stability and durability. The results of 15 pressure-release cycles that were tested at 200 kPa showed good stability and reliability of the artificial coiled yarn muscles for pressure sensation by resistance change (Figure 3f).

### 3.2. Actuation-Sensing Integration via Artificial Coiled Yarn Muscles

**Figure 4:**
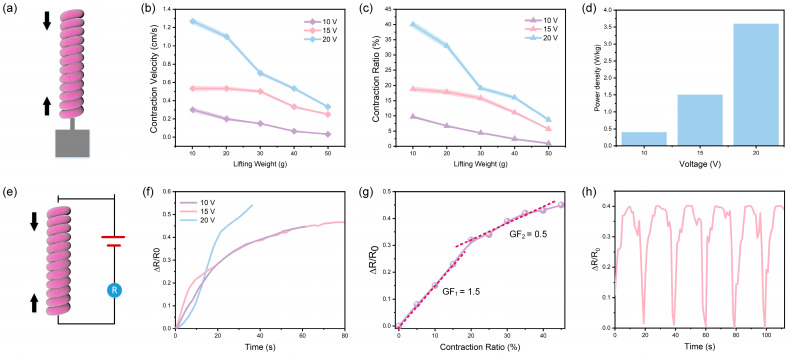
Actuation and in-situ actuation-monitoring properties. (**a**) Schematic illustration of the working mechanism for the actuation process; (**b**) The contraction velocity of the artificial muscle with different lifting weights under increasing voltages; (**c**) The contraction ratio of the artificial muscle with different lifting weights under increasing voltages; (**d**) The power density of the artificial muscle under increasing voltages; (**e**) Schematic illustration of the in situ actuation-monitoring process; (**f**) The resistance changes under different voltages with time; (**g**) The sensitivity of the contraction strain perception; (**h**) Resistance variation of actuation cycle tests.

We first investigated the actuation properties of artificial coiled yarn muscles. The actuation of the artificial coiled yarn was enabled by Joule-heating the carbon black/CNT coated layers (Figure 4a). The load from 10–50 g was applied to the muscle to stretch the muscle and separate the coils to provide space for contraction. In Figure 4b, it was observed that the contraction velocity decreases with the rising of the lifting weights and increasing with the applied voltages. In the meantime, the contraction ratio shows a negative correlation with the lifting weights and positive correlation with the applied voltages (Figure 4c). Then, we calculated the power density of different loaded weights under the same driving voltage and selected the maximum value as the power density value under this voltage (Figure 4d). As the applied voltage increases from 10 V to 20 V, the maximum power density of the muscle fibers increases from 0.4 W/kg to 3.6 W/kg.

As stated in the above section, the two-step coating and spinning process empowers the artificial muscle to be self-aware of its own actuation state. A self-developed feedback circuit is utilized to enable online detection of actuation ratio in the Joule-heating contraction process. We have built a controlling system composed of electric heating module and signal analysis module, and a custom-made software was developed for signal detection and providing feedback. Thus, the in situ monitoring of contraction ratio of the artificial coiled yarn muscles was investigated (Figure 4e). The muscle was applied with various voltages of 10 V, 15 V and 20 V, and the corresponding resistance change with time was recorded by our self-made software (Figure 4f). As the voltage increases, the time required to achieve the same change in resistance decreases. Meanwhile, larger resistance changes at higher voltages can be obtained. By corresponding time with the muscle contraction ratio, we obtained the curve of resistance change versus muscle contraction ratio. The resistance increased linearly with the muscle contraction ratio in both the low-contraction ratio region (0–20%, GF = 1.5) and the high-contraction ratio region (20–45%, GF = 0.5) but with different slopes (Figure 4g). In addition, we heated the artificial muscle in the controlling system at 20 V and conducted actuation-release cycles six times. The results also show good stability and reliability of the integrated actuation-sensing process (Figure 4h), demonstrating its promising application in monitoring its own actuation state.

### 3.3. Proof-of-Concept Demonstrations

**Figure 5:**
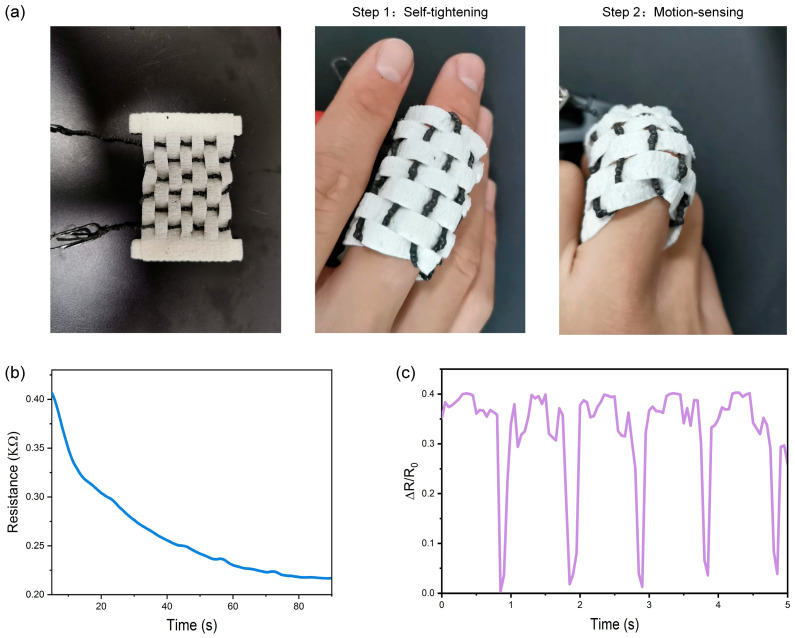
Application demonstrations of the smart textile. (**a**) The picture of the smart textile and the process of self-tightening and motion-sensing; (**b**) The resistance changes of the artificial muscle fiber in the self-tightening process under 20 V; (**c**) The resistance changes from periodic finger flexion.

In the above section, we demonstrate that the actuation strain-dependent piezoresistance properties can be used to trace the actuation path of the artificial coiled yarn muscles. Then, we fabricated smart textiles by weaving the coiled muscles into the cropped spandex, and wrap around human finger for motion-sensing (Figure 5a). To better receive the motion signal, the smart textile could generate self-tightening action when it is wrapped around the finger due to the contraction properties of the coiled yarn muscles. The resistance changes of the artificial muscle fiber in the self-tightening process under 20 V is shown in Figure 5b, which infers that the degree of tightening can be regulated by applied voltage and time to adapt to various surfaces. After the smart textile is firmly attached to the finger, the bending motion of the finger can be perceived by the resistance changes. Figure 5c shows the resistance changes from periodic finger flexion under the bending angle of 90° and demonstrates good stability and reliability of the in situ actuation sensation of the smart textile.

**Figure 6:**
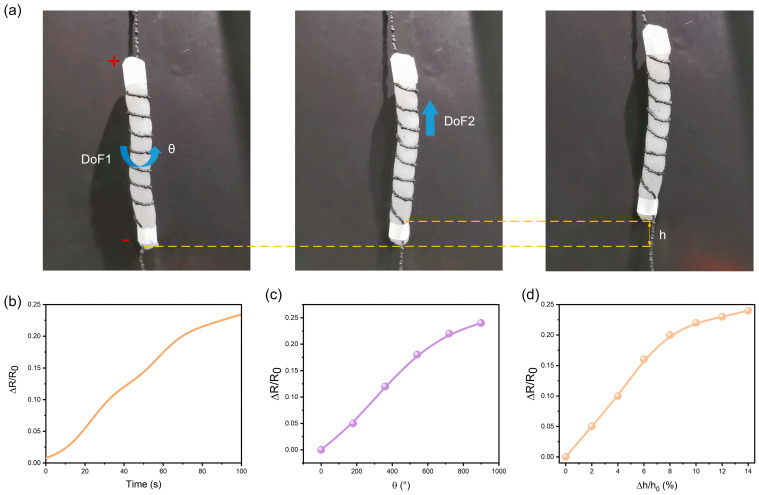
Application demonstrations of the yarn muscle entangled soft actuators. (**a**) The picture of yarn muscle entangled soft actuators and the process of twisting (DoF1) and lifting (DoF2); (**b**) The resistance changes of the artificial muscle fiber in the contraction process under 20 V; (**c**) The resistance changes of the yarn muscle with the twisting angle; (**d**) The resistance of the yarn muscle the lifting height ratio. DoF: degrees of freedom.

We further employed artificial coiled yarn muscles to enabling non-active objects with active motions and self-sensing capabilities. In Figure 6a, the artificial coiled yarn muscles were entangled with the soft tube at 30° to the longitude direction. When actuated by muscle fibers under the voltage of 20 V, torque will be generated to drive the soft tube producing rotating and lifting behaviors. The resistance changes in the lifting process were shown in Figure 6b. Meanwhile, we recorded the actuation process and corresponded to the resistance changes of the artificial muscle. The variation of rotation angle θ and the variation of lifting height h with resistance changes were obtained, as shown in Figure 6c,d, respectively. The analysis results can potentially be used to monitor the lifting height and rotation angle of the non-moving object in future.

## 4. Conclusions

Here, we have successfully demonstrated a facile approach to integrate the sensing and actuation function in one coiled muscle fiber. Through the two-step method, the conductivity and durability of the self-aware artificial coiled yarn muscles have been significantly enhanced. The resistivity is successfully reduced from 172.39 Ω·cm (first step) to 1.27 Ω·cm (second step). This artificial coiled yarn muscles can perceive multimode excitation signals (stretching strain, pressure) and then execute contractile commands in response to electrothermal stimulation, accompanied by a real-time monitoring of the actuation state. They can achieve a sensitivity (gauge factor, GF) of the contraction strain perception up to 1.5. Finally, two applications including smart textiles and entangled soft tube were conducted to demonstrate the ability to monitor and empower inactive objects with execution capabilities. The introduction of the two-step method alleviates the particle detachment problem caused by mismatched interfaces. This integrated design endows lightweight and good interface to the artificial coiled yarn muscles without compromising their flexibility, and provide promising alternatives towards the fields of wearable devices, soft robots, etc.

## Data Availability

Not applicable.

## References

[1] Marko J.F., Siggia E.D. (1996). Bending and Twisting Elasticity of DNA Volume 27, Number 4, February 14, 1994, pp 981–988. Macromolecules.

[2] Gorin A.A., Zhurkin V.B., Olson W.K. (1995). B-DNA Twisting Correlates with Base-Pair Morphology. J. Mol. Biol..

[3] Studart A.R., Erb R.M. (2014). Bioinspired Materials That Self-Shape through Programmed Microstructures. Soft Matter.

[4] Goriely A., Neukirch S. (2006). Mechanics of Climbing and Attachment in Twining Plants. Phys. Rev. Lett..

[5] Schilthuizen M., Davison A. (2005). The Convoluted Evolution of Snail Chirality. Naturwissenschaften.

[6] Frohlich C., Buskirk R.E. (1982). Transmission and Attenuation of Vibration in Orb Spider Webs. J. Theor. Biol..

[7] Haines C.S., Lima M.D., Li N., Spinks G.M., Foroughi J., Madden J.D.W., Kim S.H., Fang S., Jung de Andrade M., Goktepe F. (2014). Artificial Muscles from Fishing Line and Sewing Thread. Science.

[8] Mu J., Jung de Andrade M., Fang S., Wang X., Gao E., Li N., Kim S.H., Wang H., Hou C., Zhang Q. (2019). Sheath-Run Artificial Muscles. Science.

[9] Tawfick S., Tang Y. (2019). Stronger Artificial Muscles, with a Twist. Science.

[10] Eggeler G., Hornbogen E., Yawny A., Heckmann A., Wagner M. (2004). Structural and Functional Fatigue of NiTi Shape Memory Alloys. Mater. Sci. Eng. A.

[11] Zhou X., Fang S., Leng X., Liu Z., Baughman R.H. (2021). The Power of Fiber Twist. Acc. Chem. Res..

[12] Mirvakili S.M., Hunter I.W. (2018). Artificial Muscles: Mechanisms, Applications, and Challenges. Adv. Mater..

[13] Li M., Pal A., Aghakhani A., Pena-Francesch A., Sitti M. (2022). Soft Actuators for Real-World Applications. Nat. Rev. Mater..

[14] Wang J., Gao D., Lee P.S. (2021). Recent Progress in Artificial Muscles for Interactive Soft Robotics. Adv. Mater..

[15] Zou M., Li S., Hu X., Leng X., Wang R., Zhou X., Liu Z. (2021). Progresses in Tensile, Torsional, and Multifunctional Soft Actuators. Adv. Funct. Mater..

[16] Wang Y., Sun J., Liao W., Yang Z. (2022). Liquid Crystal Elastomer Twist Fibers toward Rotating Microengines. Adv. Mater..

[17] Ren L., Li B., Wei G., Wang K., Song Z., Wei Y., Ren L., Liu Q. (2021). Biology and Bioinspiration of Soft Robotics: Actuation, Sensing, and System Integration. iScience.

[18] Li S., Bai H., Liu Z., Zhang X., Huang C., Wiesner L.W., Silberstein M., Shepherd R.F. (2021). Digital Light Processing of Liquid Crystal Elastomers for Self-Sensing Artificial Muscles. Sci. Adv..

[19] Leng X., Zhou X., Liu J., Xiao Y., Sun J., Li Y., Liu Z. (2021). Tuning the Reversibility of Hair Artificial Muscles by Disulfide Cross-Linking for Sensors, Switches, and Soft Robotics. Mater. Horiz..

[20] Truby R.L., Wehner M., Grosskopf A.K., Vogt D.M., Uzel S.G.M., Wood R.J., Lewis J.A. (2018). Soft Robotics: Soft Somatosensitive Actuators via Embedded 3D Printing (Adv. Mater. 15/2018). Adv. Mater..

[21] Zhao H., O’Brien K., Li S., Shepherd R.F. (2016). Optoelectronically Innervated Soft Prosthetic Hand via Stretchable Optical Waveguides. Sci. Robot..

[22] Lo C.Y., Zhao Y., Kim C., Alsaid Y., Khodambashi R., Peet M., Fisher R., Marvi H., Berman S., Aukes D. (2021). Highly Stretchable Self-Sensing Actuator Based on Conductive Photothermally-Responsive Hydrogel. Mater. Today.

[23] Chen D., Liu Q., Han Z., Zhang J., Song H., Wang K., Song Z., Wen S., Zhou Y., Yan C. (2020). 4D Printing Strain Self-Sensing and Temperature Self-Sensing Integrated Sensor–Actuator with Bioinspired Gradient Gaps. Adv. Sci..

[24] Amjadi M., Sitti M. (2018). Self-Sensing Paper Actuators Based on Graphite-Carbon Nanotube Hybrid Films. Adv. Sci..

[25] Yu Y., Li L., Liu E., Han X., Wang J., Xie Y.X., Lu C. (2022). Light-Driven Core-Shell Fiber Actuator Based on Carbon Nanotubes/Liquid Crystal Elastomer for Artificial Muscle and Phototropic Locomotion. Carbon.

[26] Dong L., Ren M., Wang Y., Wang G., Zhang S., Wei X., He J., Cui B., Zhao Y., Xu P. (2022). Artificial Neuromuscular Fibers by Multilayered Coaxial Integration with Dynamic Adaption. Sci. Adv..

[27] Dong L., Ren M., Wang Y., Qiao J., Wu Y., He J., Wei X., Di J., Li Q. (2021). Self-Sensing Coaxial Muscle Fibers with Bi-Lengthwise Actuation. Mater. Horiz..

[28] Dong W., Li W., Wang K., Luo Z., Sheng D. (2020). Self-Sensing Capabilities of Cement-Based Sensor with Layer-Distributed Conductive Rubber Fibres. Sens. Actuators A Phys..

[29] Rossiter J. (2020). Spinning Artificial Spiderwebs. Sci. Robot..

[30] Zhang Y.L., Li J.C., Zhou H., Liu Y.Q., Han D.D., Sun H.B. (2021). Electro-Responsive Actuators Based on Graphene. Innovation.

[31] Wang R., Shen Y., Qian D., Sun J., Zhou X., Wang W., Liu Z. (2020). Tensile and Torsional Elastomer Fiber Artificial Muscle by Entropic Elasticity with Thermo-Piezoresistive Sensing of Strain and Rotation by a Single Electric Signal. Mater. Horiz..

